# Early onset of renal oxidative stress in small for gestational age newborn pigs

**DOI:** 10.1080/13510002.2019.1596429

**Published:** 2019-03-23

**Authors:** Hitesh Soni, Taisiya Yakimkova, Anberitha T. Matthews, Paul K. Amartey, Robert W. Read, Randal K. Buddington, Adebowale Adebiyi

**Affiliations:** a Department of Physiology, University of Tennessee Health Science Center, Memphis, TN, USA; b School of Health Studies, University of Memphis, Memphis, TN, USA; c Department of Biological Sciences, University of Memphis, Memphis, TN, USA; d College of Nursing, University of Tennessee Health Science Center, Memphis, TN, USA

**Keywords:** Kidney, oxidative stress, newborn pigs, small for gestational age, NADPH oxidase, growth restriction

## Abstract

**Objective:** Oxidative stress, a common feature in cardiovascular and renal disease is associated with the causes and consequences of fetal growth restriction. Hence, renal redox status is likely an early *determinant* of morbidity in small-for-gestational-age (SGA) infants. In this study, we examined renal oxidative stress in naturally-farrowed SGA newborn pigs.

**Methods:** We studied SGA newborn pigs with 52% less body weight and 59% higher brain/liver weight ratio compared with their appropriate-for-gestational-age (AGA) counterparts.

**Results:** The kidneys of the SGA newborn pigs weighed 56% less than the AGA group. The glomerular cross-sectional area was also smaller in the SGA group. SGA newborn pigs exhibited increased renal lipid peroxidation, reduced kidney and urine total antioxidant capacity, and increased renal nitrotyrosine immunostaining. Whereas the protein expression level of NADPH oxidase (NOX)2 was unchanged, NOX4 expression was significantly higher in SGA kidneys. The level of serum potassium was lower, but serum sodium and creatinine were similar in SGA compared with AGA newborn pigs. The serum concentrations of C‐reactive protein and NGAL, the biomarkers of inflammation and early acute kidney injury were significantly elevated in the SGA group.

**Conclusion:** Early induction of oxidative stress may contribute to the onset of kidney injury in growth-restricted infants.

## Introduction

Low birth weight due to premature birth or intrauterine growth restriction (IUGR) is associated with infant and adult cardiovascular, metabolic, and kidney disorders [1–4]. Small-for-gestational-age (SGA) newborns exhibit nephron deficit, which may disrupt renal hemodynamics and contribute to proteinuria and elevated blood pressure [3,4]. Epidemiological studies and animal experimentations have also demonstrated that SGA infants are at higher risks of developing diabetes, coronary heart disease, chronic kidney disease (CKD), and hypertension in later life [1–5]. Hence, elucidation of the mechanisms that underlie progressive organ derangements in SGA infants is necessary to reduce the burden of infant and adult cardiovascular morbidity and mortality.

Increased reactive oxygen species (ROS) generation from the mitochondria, endoplasmic reticulum, and nicotinamide adenine dinucleotide phosphate (NADPH) oxidases (NOX) have been implicated in intrauterine perturbations, such as placental insufficiency that may result in IUGR [6–9]. ROS accumulation promotes trophoblast apoptosis and autophagy and damage to placental vasculature and tissues [10]. Maternal administration of antioxidants protected against IUGR in rodents [11–14]. The levels of antioxidants were reduced, whereas, oxidants were increased in the cord blood of human SGA newborns [15–18]. Also, older children born at low birth weights are prone to oxidative stress [19–21]. Chronic treatment with the free radical scavenger TEMPOL reversed elevated arterial pressure in male [22], while antioxidant resveratrol promoted recovery from ischemia/reperfusion-induced myocardial injury in both male and female growth-restricted rat offspring [23,24]. These studies indicate that oxidative stress contributes to the etiology and consequences of IUGR. However, it remains unclear whether basal redox status is altered in naturally-occurring SGA fetal or newborn kidneys.

Human and pig newborn kidneys are comparable in size, structure, and function [25,26]. Runt pigs reflect full-term growth-restricted human neonates as they are naturally farrowed and can result from uteroplacental dysfunction, imbalanced maternal-fetal nutrient supply, or multifetal pregnancy [27–30]. In the present study, we examined renal oxidative status in full-term SGA newborn pigs.

## Materials and methods

### Animals and sample collection

Animal protocols used in this study were approved by the University of Memphis and University of Tennessee Health Science Center (UTHSC) Institutional Animal Care and Use Committees. Term vaginally-delivered newborn pigs were collected from a commercial facility with a consistent mixed strain genetic lineage. SGA (runt) pigs were selected from multiple litters based on body weights estimated to be 50% lower than littermates. Additional pigs of appropriate body weight (AGA; ∼1.6 kg) were collected from the same litters and served as controls.

Within ∼12 h after delivery, the pigs were sedated with Telazol (5 mg/kg) and then anesthetized (isoflurane, 5%) for the collection of blood by cardiac puncture and subsequent euthanasia (Euthasol; 1 ml/4.5 kg, IC). Urine was collected directly from the bladder post-mortem, and both kidneys were harvested. Serum and urine samples were stored at -80 C.

### Renal oxidative stress determination

Lipid peroxidation in the kidneys was evaluated with the thiobarbituric acid reactive substances (TBARS) kit (Cayman Chemical; Ann Arbor MI, USA; catalog number 700870). Malondialdehyde (MDA) levels were measured in kidney samples that were homogenized in RIPA buffer as we have previously described [31]. The data were normalized to protein concentrations. Urine and kidney total antioxidant capacity was determined using the Cayman Chemical’s antioxidant assay kit (catalog number 709001). Total kidney antioxidant capacity was also normalized to protein concentrations. Nitrotyrosine was immunostained in kidney sections with a rabbit polyclonal antibody (Abcam Inc. Cambridge, MA; ab42789). Images were acquired from randomly-selected fields using a Zeiss LSM 710 confocal microscope and were analyzed using the ImageJ software (NIH, Bethesda, MD USA).

### Western blot

SDS-polyacrylamide gel electrophoresis was performed as we have previously described [31–33]. Briefly, proteins were separated by 4–20% ExpressPlus PAGE Gel (GenScript Corporation, Piscataway, NJ) in a Mini Trans-Blot Cell (Bio-Rad) and transferred onto PVDF membranes using a Pierce Fast Semi-Dry Blotter (Life Technologies, Grand Island, NY, USA). Immunoreactive protein blots were visualized and documented using a gel documentation system (Bio-Rad, Hercules, CA). Protein band intensities (normalized to beta-actin) were analyzed by digital densitometry (Quantity One software; Bio-Rad). NOX2 (ab129068) and NOX4 (ab133303) antibodies were purchased from Abcam.

### Serum electrolytes and biomarker assays

Serum concentrations of sodium and potassium were quantified using the fully-automated X•pedite ion-selective electrode veterinary electrolyte analyzer (DiaSys Diagnostic Systems, USA, LLC; Wixom, MI). The serum level of nitrogenous waste product creatinine was determined at the UTHSC Regional Biocontainment Lab using the respons 910 veterinary chemistry analyzer (DiaSys). All analyses were performed following manufacturers’ instructions. Serum concentrations of neutrophil gelatinase-associated lipocalin (NGAL) and C-reactive protein (CRP) were quantified using porcine-specific NGAL (Abcam, Cambridge UK; catalog number ab207924) and CRP (Immunology Consultants Laboratory, Portland OR USA; catalog number E-5CRP) ELISA kits.

### Histology

Formalin-fixed kidney samples were processed into paraffin, cut at 5 µm, and stained with hematoxylin and eosin (H&E) and Periodic acid-Schiff (PAS) kits in a commercial lab (Mass Histology Services, Worcester, MA). The samples were evaluated for potential differences in side-by-side comparison by a certified veterinary pathologist. Both H&E- and PAS-stained slides were evaluated in comparison of SGA and AGA renal histology. The sections were imaged using a Nikon Ci microscope, 20x Plan APO objective, Fi2 camera (2560 × 1920-pixel jpeg images) and Nikon NIS Elements software. Image calibration was performed with a stage micrometer and glomeruli were measured using Adobe PhotoShop software. Between the outer immature cortex and the medulla, 7 random images were collected per section and all recognizable glomeruli were measured unless they touched image margins or were tangential. To measure the approximate average cross-sectional area, the largest 5 values were excluded as outliers and the next largest 25 values, in each group, were compared.

### Data analysis

Statistical analysis was performed using the GraphPad InStat statistics software (Graph Pad, Sacramento, CA). Data were compared using the Student’s *t*-tests for paired or unpaired data and analysis of variance with the Student-Newman-Keuls test for multiple comparisons. All data are reported as mean ± standard error of mean (SEM). Differences between data sets were considered significant when the *P value is less than* 0.05.

## Results

**Figure 1.**
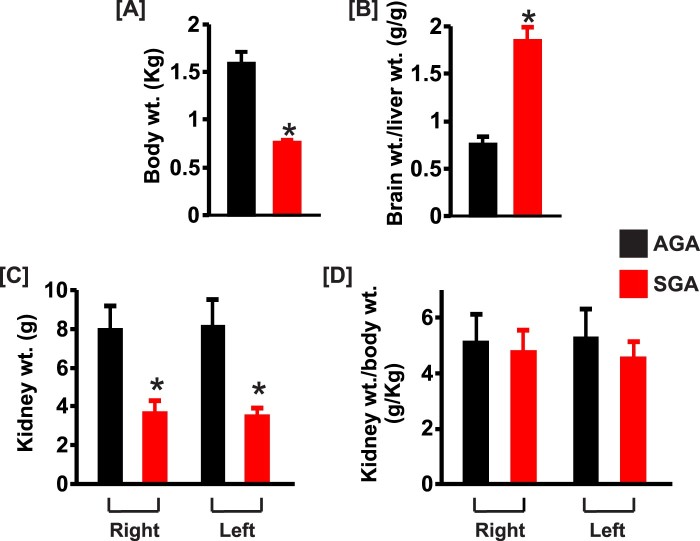
Bar graphs summarizing (a) body weights, (b) brain to liver weight ratio, (c) kidney weights, and (d) kidney to body weight ratio of AGA and SGA newborn pigs (*n* = 5 each). *P < 0.05 vs. AGA.

All SGA pigs were as active as AGA littermates, and none had any evidence of infection or respiratory distress. Figure 1 summarizes the mean body and kidney weights of AGA and SGA newborn pigs. Body weights averaged 52% lower for SGA pigs (Figure 1(a)). Mean brain to liver weight ratio was higher in the SGA group (Figure 1(b)). There were no differences in the weights of the right versus left kidneys in each group (Figure 1(c)). The weights of the right and left kidneys were ∼54% and 57% less, respectively in SGA compared with AGA newborn pigs (Figure 1(c)). However, the kidney to body weight ratio was comparable in both groups, indicating that differences in kidney weights were related to body weight variances (Figure 1(d)).

**Figure 2.**
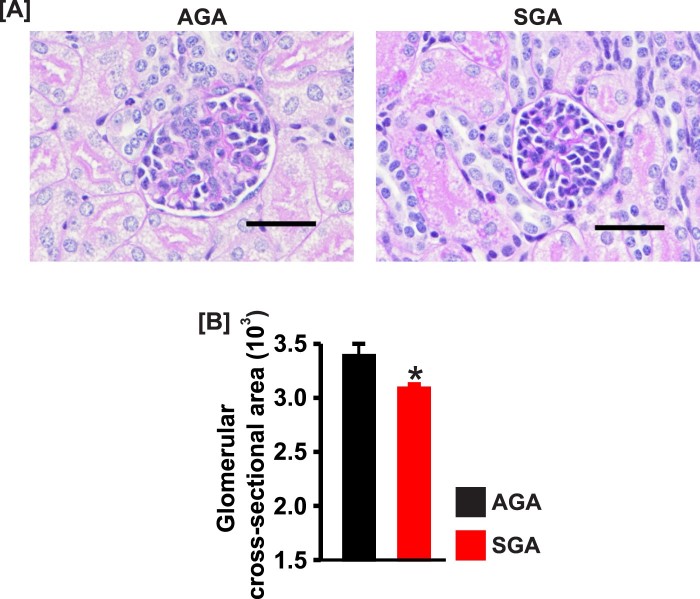
(a) Kidney section images (PAS staining) and (b) bar graphs showing the mean glomerular cross-sectional area in AGA and SGA newborn pigs (*n* = 5 each). *P < 0.05 vs. AGA; scale bar = 50 µm.

Renal histology revealed trends of slightly greater immaturity and glomerular hypercellularity in the SGA group. The SGA group also showed marginally thicker immature cortex, and the AGA group showed somewhat taller epithelium in the collecting ducts. However, the two groups overlap broadly, and these features were not unique discriminators. There was no evidence of glomerular or tubular damage in AGA versus SGA pig kidneys (Figure 2(a)). However, the glomerular cross-section area was smaller (∼9%) in SGA newborn pigs (Figure 2(a) and (b)).

**Figure 3.**
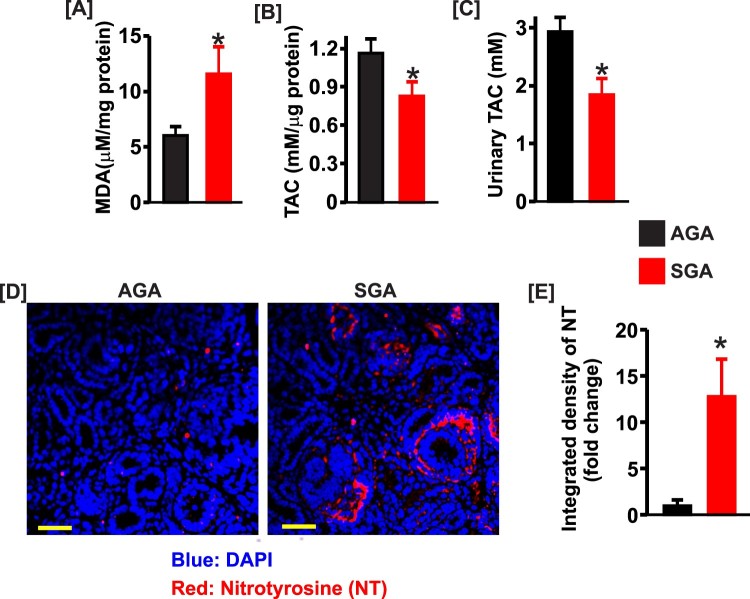
Bar graphs summarizing (a) the levels of malondialdehyde (MDA; *n* = 5 each), (b) kidney total antioxidant capacity (*n* = 5 each), and (c) urine total antioxidant capacity (*n* = 4 each) in SGA compared with AGA newborn pigs. (d) Confocal microscopy images showing immunostaining of nitrotyrosine in AGA and SGA newborn pig kidney sections. (e) bar graphs of mean fluorescence density in AGA and SGA newborn pig kidney sections immunostained for nitrotyrosine (NT); *P < 0.05 vs. AGA. Scale bar = 50 µm.

The lipid peroxidation product, MDA was increased ∼ 2-fold in SGA kidney lysates (Figure 3(a)). To further evaluate the redox status of the newborn pig kidneys, we determined the renal total antioxidant capacity, a measure of the cumulative effect of antioxidants [34]. As shown in Figure 3(b) and (c), the kidney and urine total antioxidant capacity was significantly reduced in SGA pigs. Immunofluorescence staining indicated that nitrotyrosine, a marker of peroxynitrite, was essentially absent in AGA kidney sections (Figure 3(d) and (e)). However, SGA kidneys showed robust immunostaining for nitrotyrosine (Figure 3(d) and (e)).

**Figure 4.**
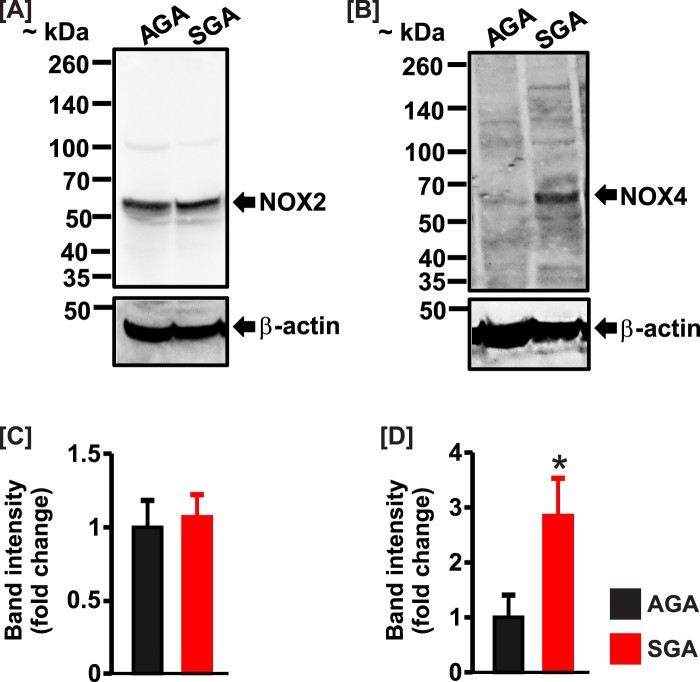
(a) and (b) Western blot images and (c) and (d) bar graphs demonstrating protein expression levels of NOX2 and NOX4 in the kidneys of AGA (*n* = 4) and SGA (*n* = 5) newborn pigs. Data were normalized to AGA; *P < 0.05 vs. AGA.

NOX2 and 4 are major sources of ROS in the kidney [35]. Hence, we investigated whether the expression levels of the enzymes are altered in the kidneys of SGA newborns. Western blotting indicated that NOX2 was unchanged; whereas, NOX4 expression was increased ∼3-fold in kidney samples of SGA compared with AGA pigs (Figure 4).

**Figure 5.**
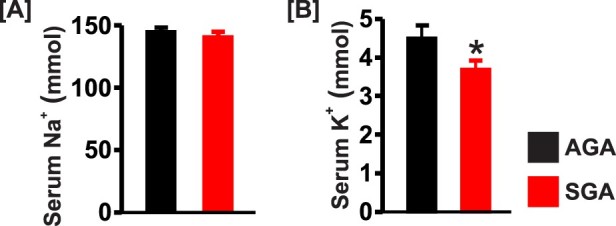
Bar graphs summarizing serum concentrations of (a) sodium, (b) potassium in AGA and SGA newborn pigs (*n* = 5 each); *P < 0.05 vs. AGA.

**Figure 6.**
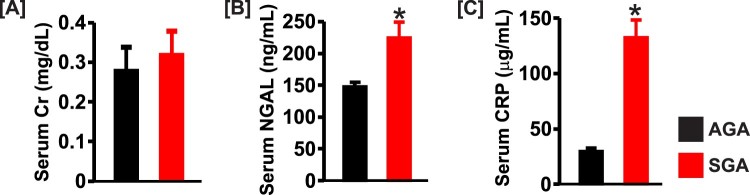
Bar graphs summarizing serum levels of (a) creatinine (Cr), (b) NGAL, and (c) C-reactive protein (CRP) in AGA and SGA newborn pigs (*n* = 5 each); *P < 0.05 vs. AGA.

Serum sodium concentration was similar in both groups, but the level of serum potassium was lower in the SGA newborn pigs (Figure 5(a) and (b)). Serum creatinine was slightly higher in SGA pigs but did not reach statistical significance (Figure 6(a)). By contrast, serum concentrations of NGAL and CRP were significantly elevated ∼1.5-fold and 4-fold, respectively in the SGA group (Figure 6(b) and (c)).

## Discussion

We used naturally occurring growth-restricted newborn pigs to investigate basal renal oxidative status in SGA newborns. We show that the average kidney weight of the SGA newborn pigs was about half of the AGA group and related to body weight differences. Furthermore, the SGA newborn pigs exhibited higher renal lipid peroxidation, lower renal antioxidant capacity, and higher expression levels of renal nitrotyrosine and NOX4. Moreover, the serum concentrations of inflammation and kidney injury biomarkers CRP and NGAL were significantly higher in SGA when compared with AGA newborn pigs. Our data suggest that early oxidative stress may contribute to the onset of kidney injury in SGA infants. As the pigs were littermates, the differences are attributed to the consequences of being SGA.

Previous morphometric analyses indicated that runt pigs display similar characteristics to human asymmetric IUGR with a hallmark of increased mean brain to liver weight ratio [36], a fact corroborated in this study. Growth-restricted pigs also exhibited nephron deficits and impaired glomerular filtration rate (GFR) [36,37]. Here, we demonstrate that the mean glomerular cross-sectional area was slightly, but significantly lower in SGA compared with AGA newborn pigs. Although a reduction in nephron numbers may result in compensatory glomerular hypertrophy as SGA neonates mature [38], nephron and filtration surface area deficits may contribute to a decline in glomerular filtration within the first week of life of the SGA newborns. Morphological and functional changes in the immature SGA kidneys may promote local stressors and set the stage for an unfavorable course of cardiovascular and renal disease.

ROS, including superoxide anion, hydroxyl radical, and hydrogen peroxide are produced by several mechanisms, including cellular respiration and enzymatic reactions [39,40]. Although at low levels, endogenously generated ROS are involved in cellular signaling mechanisms that regulate homeostasis. Amplified ROS production overwhelms antioxidant defense systems resulting in oxidative stress [39,40]. Oxidative stress induces cellular injury and plays a significant role in the pathophysiology of cardiovascular disease, including hypertension, atherosclerosis, myocardial infarction, and congestive heart failure [40,41]. Increased generation of reactive oxygen and nitrogen species in the kidney dysregulates renal hemodynamics and induces renal cell death [31,42–45]. Redox-mediated renal insults may also be involved in the initiation of systemic hypertension [44,46,47]. To prevent oxidative stress, cellular redox state is fine-tuned during fetal growth [48,49]. However, intrauterine stressors, including abnormal nutritional supply, prenatal hypoxia, and fetotoxic drugs may alter fetomaternal hemodynamics and fetal growth thereby engendering perinatal organ dysfunctions and their short- or long-term sequelae [48,50]. Data presented herein demonstrate elevated renal oxidative stress in SGA newborn pigs as evidenced by increased levels of renal lipid peroxidation and nitrotyrosine (an index of peroxynitrite-dependent oxidative damage), as well as attenuated kidney and urine total antioxidant capacity. This study does not elucidate specific oxyradicals and related species that are increased in the kidneys of growth-restricted newborn pigs. However, our findings indicate that renal oxidative stress manifests early in SGA infants.

The seven-member NOX family (NOX1-5 and DUOX1 and DUOX2) are key enzymes that catalyze cellular ROS-generating reactions [51,52]. NOX2 and NOX4 are the predominant isoforms in the kidney and are expressed in fibroblasts and vascular, glomerular, and tubular cells [35,53]. Upregulation of renal NOX2 or NOX4 or both have been implicated in acute kidney injury (AKI) and CKD [35,53]. Unlike NOX2, Western immunoblotting revealed that expression of NOX4, a regulator of peroxynitrite signaling [54–56], is increased in the kidneys of SGA newborn pigs. These data suggest NOX4 contributes to renal oxidative stress in the pigs. The mechanisms that trigger the increased expression of NOX4 in SGA newborn kidneys are unclear. However, studies have shown that angiotensin II (Ang II) induces NOX isoforms in a variety of tissues and organs, including kidneys which may contribute to hypertension [35,53]. Interestingly, Ang II type 1 receptor expression levels were found to be upregulated in the kidneys of SGA pigs [57]. The plasma concentration of Ang II has also been shown to be elevated in growth-restricted newborn human and lambs [58–60]. Conceivably, amplified renin-angiotensin system controls renal NOX expression and activity in SGA infants.

Growth-restricted newborns exhibit electrolyte imbalance and hallmarks of AKI [61–63]. Here, we show that the serum concentration of sodium was unchanged in SGA newborn pigs, which is consistent with previously reported normal fractional sodium excretion [37]. However, the SGA newborn pigs in this study exhibited hypokalemia. Serum creatinine concentration, a poor biomarker of early stages of AKI was not altered in SGA newborn pigs. Histopathology data revealed a lack of apparent kidney damage in SGA newborn pigs, but an increase in the serum level of NGAL, an early predictor of AKI [64], suggests the onset of AKI in the pigs. Although serum and urinary NGAL are effective biomarkers of early AKI, an increase in NGAL levels can also occur in acute and chronic inflammation [65]. The elevated serum CRP observed in the SGA group indicates the presence of systemic inflammation. Moreover, CRP has not only been shown to cause kidney injury, but its circulating levels are also increased in AKI and CKD [66–68]. Since oxyradical generation and renal inflammation are both involved in the initiation and progression of kidney injury, possible pathophysiology mechanisms of early renal insults in growth-restricted newborns may include a crosstalk between renal oxidative stress and inflammation.

In summary, we provide evidence of early renal oxidative stress and kidney injury in SGA newborn pigs. As the kidneys are involved in the long-term control of homeostasis, more studies using naturally-occurring large animal models of IUGR are needed to understand progressive renal dysfunction in growth-restricted infants and possible preemptive therapeutic approaches to mitigate the development of short- and long-term cardiovascular and renal disease.
